# Effects of Molecular Length and Polarity of Chain Extenders on Microphase Separation and on Thermal and Mechanical Properties of Rigid Polyurethane Foam

**DOI:** 10.3390/polym18030355

**Published:** 2026-01-28

**Authors:** Yaonan Liu, Renchun Tian, Xinling Hao, Danning Tang, Yanchen Fang, Xihuan Liu, Mingliang Sun, Tao Zhuang

**Affiliations:** 1 Key Laboratory of Rubber-Plastics, Shandong Provincial Key Laboratory of Rubber-Plastics, Ministry of Education, School of Polymer Science and Engineering, Qingdao University of Science and Technology, Qingdao 266042, China; 13061406072@163.com (Y.L.);; 2 College of Materials Science and Engineering, Zhejiang University of Technology, Hangzhou 310023, China; 3 School of Materials Science and Engineering, Ocean University of China, Qingdao 266100, China

**Keywords:** rigid polyurethane, chain extender, hydrogen bond, molecular chain length, mechanical property

## Abstract

In this work, rigid polyurethane materials were synthesized via a one-step polymerization method using isocyanate (MDI) and polyether polyol (4110S) as the main raw materials, with 1, 4-butanediol (BDO), 1, 6-hexanediol (HDO), diethylene glycol (DEG), and dipropylene glycol (DPG) as chain extenders. The influence of chain extender structure on the mechanical properties of rigid polyurethane was systematically investigated. The results indicate that when BDO was employed as a chain extender, the polyurethane exhibited the most uniform pore size distribution and the best mechanical properties. It was found that hydrogen bonding plays a dual role: on the one hand, it promotes microphase separation between soft and hard segments; on the other hand, it extends the molecular chains’ length, which hinders segment separation and consequently constrains its mechanical properties. Further analysis reveals that the influence of molecular chain length on mechanical properties outweighs that of polarity.

## 1. Introduction

Polyurethane, a block copolymer composed of soft and hard segments, has its hardness and performance tailored for various applications by adjusting the ratio between these segments [1,2,3,4,5,6]. Generally, a rigid polyurethane foam (RPUF) with a hardness greater than Shore 75A is considered [7,8,9]. RPUF is mainly composed of polyols, mainly polyester polyols and polyether polyols, which constitute the soft segments of the polymer chains. Isocyanate participates in forming the primary hard segments within the chains, while chain extenders (typically small-molecule diols, diamines, or ethanolamine) define the length of these hard segments and regulate the phase separation between soft and hard domains [10,11,12]. Because RPUF has the characteristics of low apparent density, sound insulation, easy processing, and other characteristics, it is widely used in aerospace, automotive, construction, and other fields [13,14,15].

The main function of a chain extender is to increase molecular weight, form a hydrogen bond, and increase the chain segment length [16,17]. The structure of the chain extender affects the microphase separation of soft and hard segments and then affects the mechanical properties of polyurethane. From the perspective of system energy, microphase separation of soft and hard segments is beneficial to improving the mechanical properties of rigid polyurethane materials [18,19,20].

Therefore, in this work, 1, 4-butanediol (BDO), 1, 6 hexanediol (HDO), diethylene glycol (DEG), and dipropylene glycol (DPG) were used as chain extenders to study the effects of the structure and length of chain extenders on the mechanical and thermal properties of polyurethane.

## 2. Materials and Methods

### 2.1. Materials

**Figure 1:**
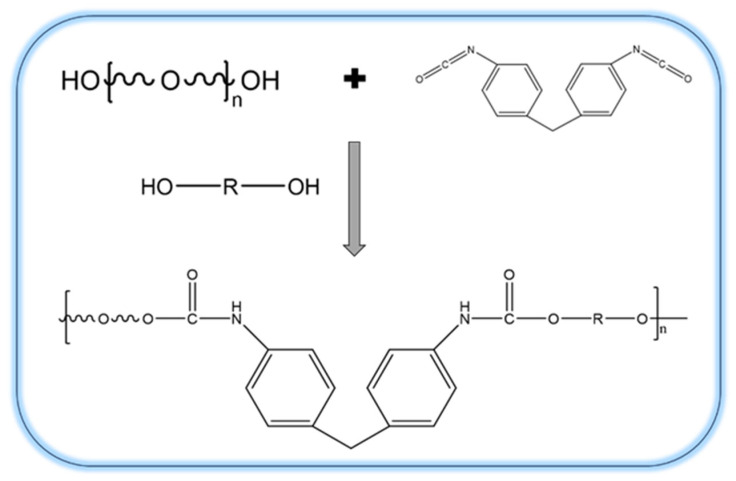
Synthesis flow of RPUF using different chain extenders.

Polyether polyol 4110S was purchased from Shandong Weishang Chemical Co., Ltd (Jining, China). Methylene diphenyl diisocyanate (MDI) was supplied by Wanhua Chemical Group Co., Ltd (Yantai, China). The chain extenders, BDO, HDO, DEG, and DPG, were procured from Shanghai Aladdin Biochemical Technology Co., Ltd (Shanghai, China). Catalyst A33 was purchased from Shandong Jiying Chemical Technology Co., Ltd., (Weifang, China) triethanolamine was obtained from Jinan Ningsheng Trading Co., Ltd., (Jinan, China) and surfactant AK-158 from Shandong Olong Chemical Co., Ltd (Jinan, China). The chemical structures of the chain extenders are summarized in Table 1, the RPUF reaction process is shown in Figure 1.

### 2.2. Preparation of Foams

**Figure 2:**
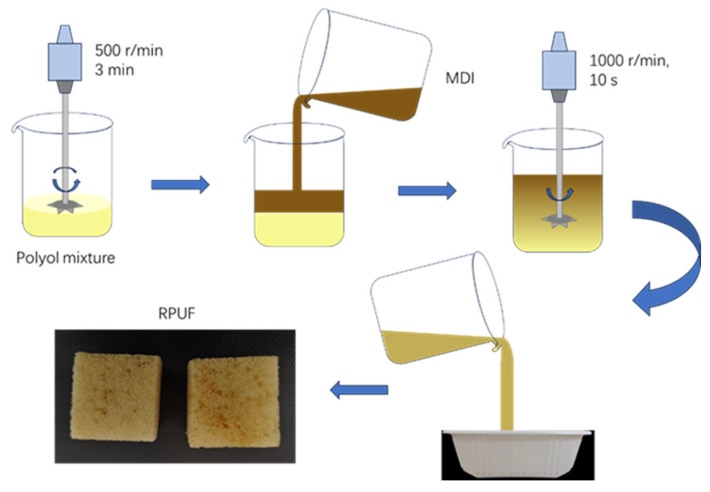
Synthesis technology of RPUF with different chain extenders.

According to the predetermined formulation (as listed in Table 2), the chain extender was added by mass based on its hydroxyl value, and the overall R-value (molar ratio of -NCO to -OH) was maintained at 1.1. The polyether polyol composition (Part A) was weighed and mixed with the respective chain extender. The mixture was stirred at 500 r/min for 3 min. Subsequently, MDI (Part B) was rapidly added to Part A, followed by vigorous stirring at 1000 r/min for 10 s. The resulting mixture was immediately poured into a mold for free-rise foaming. After the completion of foaming, the material was cured for 2 h, demolded, cut into specimens according to the standard dimensions, and conditioned for 24 h prior to performance testing. A schematic diagram of the foaming process is presented in Figure 2.

### 2.3. Characterization

Fourier transform infrared (FTIR) analysis was performed using a Bruker Vertex 70 spectrometer (Karlsruhe, Germany) over a wavenumber range of 4000~400 cm^−1^. The Differential Scanning Calorimetry Test (DSC) was conducted using a NETZSCH DSC204F1 instrument (Selb, Germany) under a nitrogen atmosphere (flow rate: 50 mL/min). The temperature program was set as follows: cooling from 40 °C to 20 °C, holding for 2 min, and then heating to 150 °C at a rate of 10 °C/min. Thermogravimetric analysis (TGA) was performed using a Mettler Toledo TGA2 instrument (Zurich, Switzerland). The measurements were conducted under a nitrogen atmosphere with a flow rate of 50 mL/min. Samples were heated from 30 °C to 650 °C at a constant heating rate of 10 °C/min. The cellular morphology of RPUF was observed with a Nikon SMZ1500 (Tokyo, Japan) stereoscopic microscope at 20× magnification. The viscosity of the polyol mixture (Part A) containing different chain extenders was measured at 25 °C using a Brookfield (Toronto, ON, Canada) DV-II+ Pro rotational viscometer.

The mechanical properties were evaluated according to GB/T 8813-2020 [20] (compression) and GB/T 9341-2008 [21] (flexural) standards using a Zwick Z010 universal testing machine (Ulm, Germany). The crosshead speeds were set to 10 mm/min for compression tests and 5 mm/min for three-point bending tests [21,22].

## 3. Results and Discussion

### 3.1. FTIR Analysis

During the synthesis of polyurethane, the chain extender is primarily incorporated into the main chain by reacting its hydroxyl groups with isocyanate groups. Typically, a low-molecular-weight diol serves as the chain extender, where the -OH groups react with -NCO groups to form urethane linkages (-NHCOO-). The reaction mechanism is illustrated in Figure 1.

**Figure 3:**
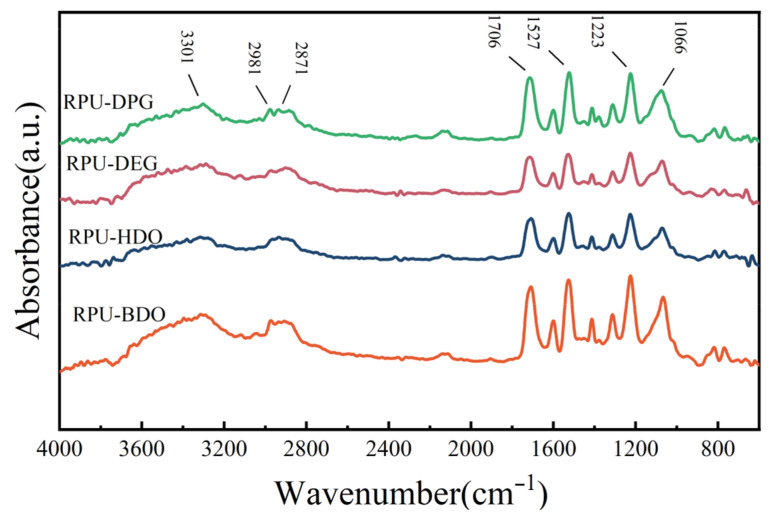
Infrared spectra of four RPUFs.

FTIR spectroscopy was employed to confirm the chemical structure. The spectrum shows characteristic absorption bands corresponding to the formed urethane bonds (as shown in Figure 3): the N-H stretching vibration at 3301 cm^−1^ [23], asymmetric and symmetrical and symmetric C-H stretching vibration at 2981 cm^−1^ and 2871 cm^−1^, respectively, and the C=O stretching vibration at 1706 cm^−1^. Additionally, the C-N stretching vibration in the carbamate group appears at 1527 cm^−1^, the C-O stretching vibration in the urethane ester group is observed at 1223 cm^−1^, and the hydrogen-bonded C-O stretching vibration is present at 1066 cm^−1^ [24,25]. These spectral features confirm the successful synthesis of the polyether-based RPUF.

### 3.2. Thermal Properties

**Figure 4:**
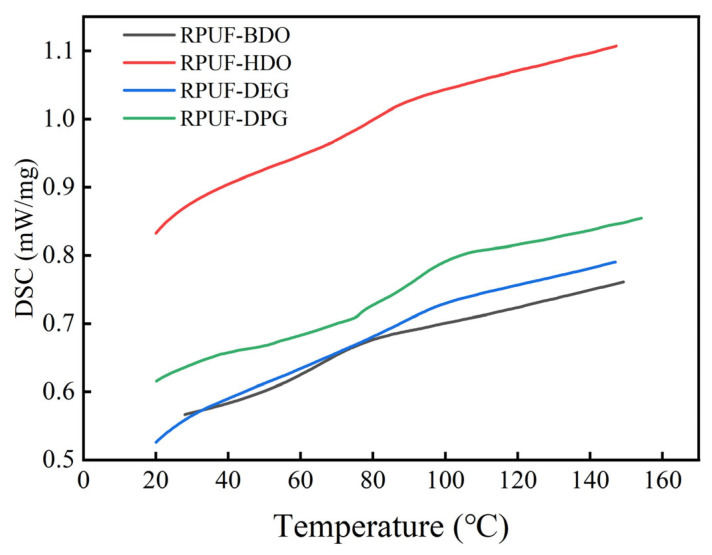
DSC Measurement Curve of four RPUFs.

The glass transition temperature (Tg) of the RPUF, as summarized in Table 3 and Figure 4, varies significantly with the choice of chain extender. RPUF-BDO exhibits the lowest Tg, which is attributed to the high structural regularity of BDO. This regularity promotes the ordering and aggregation of hard segments, leading to a higher degree of microphase separation between the hard and soft domains. Consequently, the constraint exerted by the hard segment on the soft segments is reduced, allowing for greater soft-segment mobility and a lower Tg.

In contrast, the longer chain of HDO impedes the effective packing and aggregation of hard segments, resulting in a lower degree of microphase separation. This enhanced mixing strengthens the interaction between hard and soft segments, thereby restricting soft-segment mobility and yielding a higher Tg for RPUF-HDO.

RPUF-DPG demonstrates the highest Tg among the series. This is primarily due to the presence of flexible ether linkages (-O-) and the longer molecular chain of DPG, which collectively hinder the aggregation of hard segments and maintain a low degree of microphase separation. Furthermore, the strong interchain interactions associated with DPG significantly enhance the restraining effect of the hard segment on the soft-segment matrix. This severely limits the mobility of the soft segment, leading to the observed elevation in T_g_.

**Figure 5:**
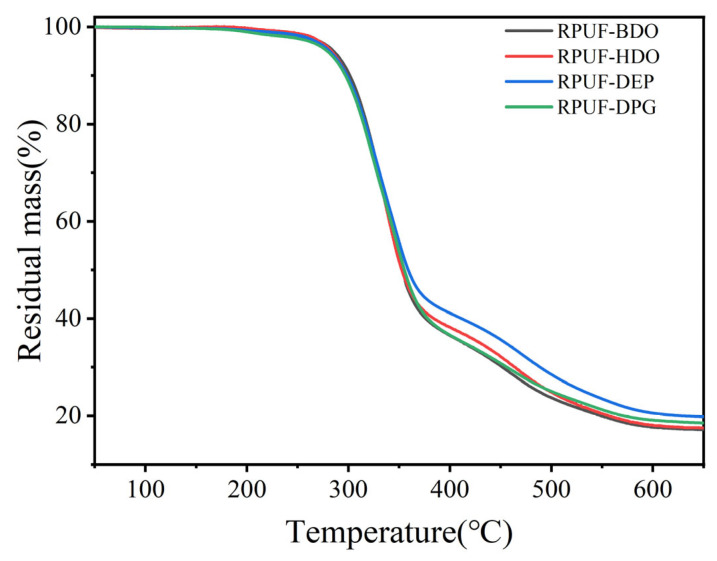
Thermal analysis of four RPUFs.

As shown in Figure 5, thermogravimetric analysis reveals that all RPUFs display an initial minor mass loss around 250 °C, followed by a rapid degradation step beginning near 300 °C. An inflection point appears at approximately 400 °C, leading to a secondary stage of rapid mass loss at about 450 °C. This indicates that the type of chain extender has a relatively limited influence on the overall thermal stability of the RPUFs, primarily causing slight variations in their high-temperature degradation pathways. The marginally higher final char yield observed for the samples containing DEG and DPG can be attributed to cyclization reactions occurring in the later stages of decomposition. Specifically, the flexible ether linkages present in DEG and DPG facilitate cyclization at elevated temperatures, promoting carbonaceous structure formation, reducing volatile release, and thereby increasing the residual char content. In contrast, RPUFs modified with BDO and HDO exhibit less pronounced cyclization, resulting in comparatively lower char residues.

### 3.3. Cell Morphology

**Figure 6:**
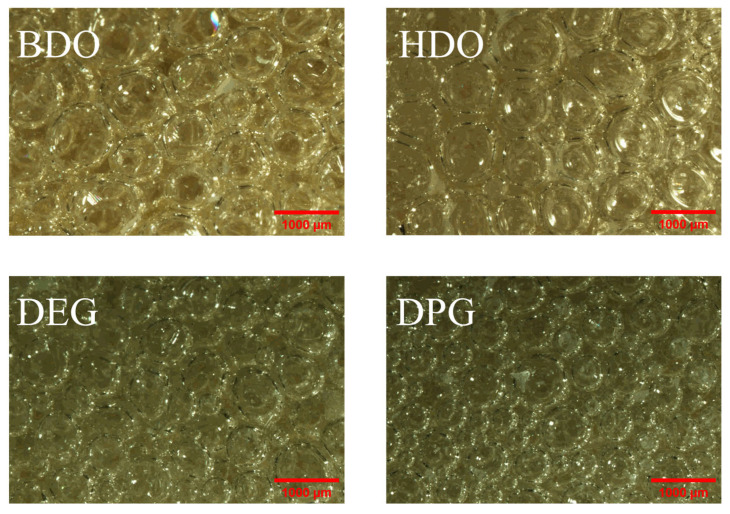
Cell morphology of four RPUFs.

**Figure 7:**
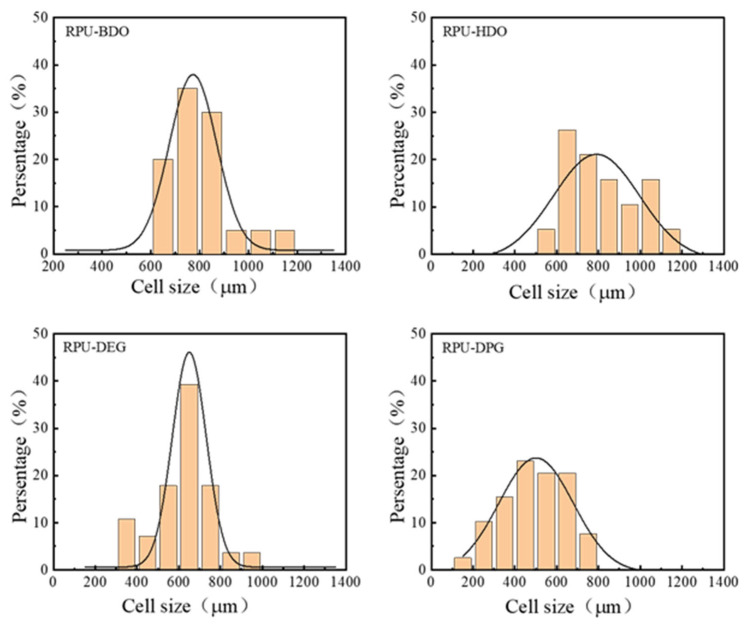
Cell size distribution of four RPUFs.

The cellular structure of the RPUF was observed with a stereoscopic microscope at a 10× eyepiece and a 2× objective lens, as shown in Figure 6. To quantitatively analyze the cellular structure, the cell diameter of approximately 100 pores per sample was measured to obtain the pore size distribution, as shown in Figure 7. Combined with the morphological observations in Figure 5, these results indicate that the RPUF prepared with DPG as a chain extender possesses the smallest average cell diameter, whereas the foam formulated with BDO exhibits the largest.

This variation in cell morphology can be attributed to differences in the viscosity of the chain extenders and their impact on the system during foaming. The intrinsic viscosities of the chain extenders differ significantly, with measured values at 25 °C being 66 mPa·s for BDO, 78 mPa·s for HDO, 17 mPa·s for DEG, and 19 mPa·s for DPG. DPG has the highest intrinsic viscosity, while BDO has the lowest. When incorporated into Part A, the higher viscosity of DPG significantly increases the overall viscosity of the reactive mixture. Concurrently, the viscosity of the greater molecular weight of DPG further contributes to system viscosity as polymerization proceeds.

During polyurethane chain growth and foam formation, the system formulated with DPG experiences a more pronounced increase in system viscosity. This elevated viscosity strongly restricts bubble expansion, ultimately resulting in the formation of smaller cells. In contrast, the lower viscosity and molecular weight of BDO lead to reduced resistance to bubble growth, thereby yielding foam with a larger average cell diameter.

The cell size distribution is another critical factor influencing the mechanical properties of polyurethane foam. As shown in Figure 6, the foam prepared with BDO exhibits the narrowest cell size distribution, ranging from 528 μm to 1172 μm. A narrower t distribution promotes more uniform stress transfer under loading: external forces are distributed more evenly across adjacent cells, reducing local stress concentration and delaying cell wall rupture. This results in an enhanced ability to withstand higher external loads.

### 3.4. Apparent Density

**Figure 8:**
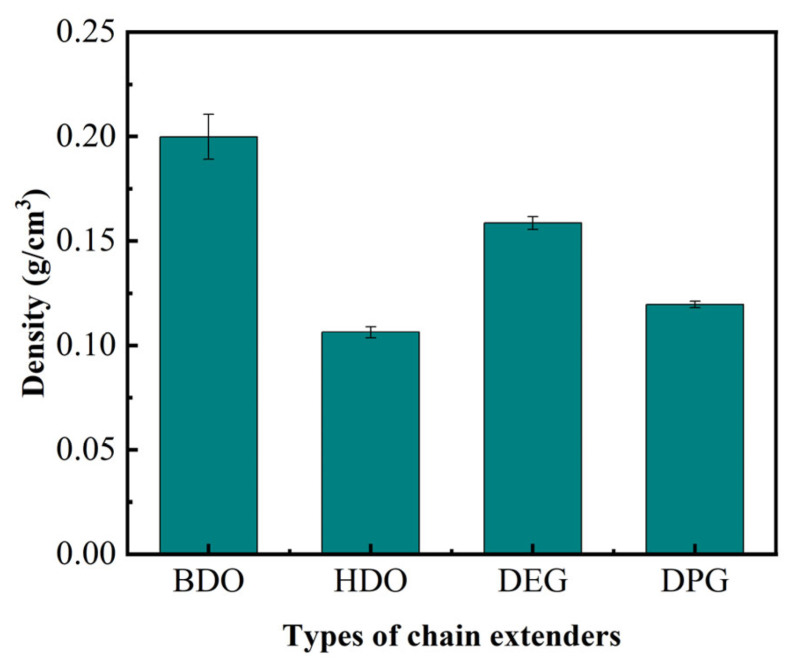
The effect of chain extenders on the apparent density of RPUF.

Apparent density serves as a critical parameter for controlling the mechanical properties of closed-cell RPUFs. As shown in Figure 8, the density of RPUF decreases with an increase in the molecular chain length of the chain extender. When a polar group (–O–) is introduced into the chain extender, the influence of molecular chain length on density becomes more pronounced than that of the polar group itself. Using BDO as the chain extender results in a highly regular molecular structure, which facilitates the formation of a densely packed arrangement. This leads to a greater number of molecular chains per unit volume, a higher mass, and consequently the highest density among the series (RPUF-BDO). In contrast, the presence of the ether linkage (–O–) in DEG reduces structural regularity and introduces steric hindrance, hindering efficient chain packing. Thus, fewer molecular chains occupy a given volume, the mass is lower, and the density of RPUF-DPG is reduced. When DPG is employed as a chain extender, its longer molecular backbone results in the lowest packing efficiency, giving RPUF-DPG the lowest density of all the foams studied.

### 3.5. Mechanical Properties

**Figure 9:**
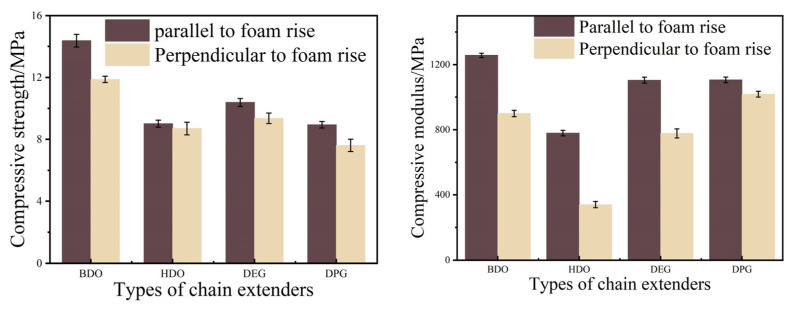
Specific compressive properties of RPUF.

**Figure 10:**
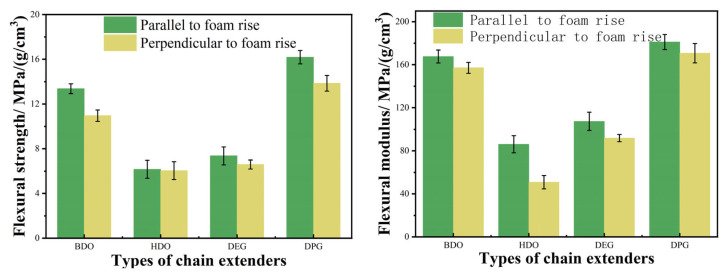
Specific flexural properties of RPUF as a function of chain extender structure.

Specific strength and specific modulus, defined as strength and stiffness per unit density, respectively, are fundamental parameters for selecting materials in lightweight applications. As shown in Figure 9 and Figure 10, the RPUF chain extended with BDO demonstrated superior specific compressive and flexural properties compared to those extended with HDO. Correspondingly, DPG-derived RPUF exhibited higher specific compressive and flexural properties than their DEG-derived counterparts. Among all evaluated systems, the foam with BDO demonstrated the optimal overall mechanical properties, with those based on DPG, DEO, and HDO following in sequence.

These mechanical trends can be interpreted based on the molecular structures of the respective chain extenders. The high structural regularity of BDO facilitates crystallization within the hard segments, thereby enhancing stiffness. However, this also results in greater brittleness, leading to lower flexural strength. In contrast, the more flexible molecular chains of DPG impart enhanced ductility to the hard segments, allowing RPUF-DPG to achieve higher flexural strength while maintaining a comparable modulus. The excellent mechanical properties of the BDO-based foam originate from key structural features—a short molecular chain and high symmetry—thereby enabling the ordered arrangement and dense packing of hard segments [25]. The short hard segments from MDI promote intermolecular hydrogen bonding, which in turn restricts the penetration of soft segments into the hard domains, thereby enhancing microphase separation. Furthermore, the BDO-based foam exhibits the narrowest cell size distribution; this morphology promotes uniform stress transfer under load and contributes to its improved mechanical performance.

## 4. Conclusions

In this work, BDO, HDO, DEG, and DPG were employed as chain extenders to study the effects of the structure and length of chain extenders on the mechanical and thermal properties of polyurethane. The results indicate that:

1. The longer the molecular chain, the lower the degree of microphase separation, the stronger the binding ability of the hard segment on the soft segment, the more difficult the movement of the soft segment, and the higher the Tg.
2. For RPUF-BDO and RPUF-DEG samples, the effect of increasing hydrogen bond content on the degree of microphase separation in the soft and hard segments is smaller than that of molecular chain length.
3. When BDO is added to polyurethane as the chain extender, the dense packing of molecular chains and the hydrogen bond formed between them improve the microphase separation degree of soft and hard segments, thereby imparting the optimal overall performance to the material.

## Figures and Tables

**Table 1:** Structure of chain extender.

Chain Extender	Abbreviation	Chemical Construction
butane-1,4-diol	BDO	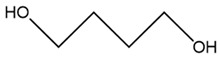
hexane-1,6-diol	HDO	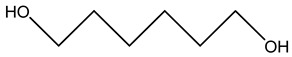
2,2′-oxybis(ethan-1-ol)	DEG	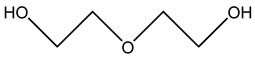
3,3′-oxybis(propan-1-ol)	DPG	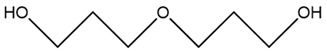

**Table 2:** Raw material composition.

Chain Extender	RPUF-BDO	RPUF-HDO	RPUF-DEG	RPUF-DPG
4110S	50	50	50	50
BDO	8	0	0	0
HDO	0	20.98	0	0
DEG	0	0	18.84	0
DPG	0	0	0	23.82
H2O	1	1	1	1
A33	0.5	0.5	0.5	0.5
A158	0.5	0.5	0.5	0.5
MDI	187.02	187.02	187.02	187.02

**Table 3:** Glass transition temperature (Tg) of four RPUFs.

Sample	RPUF-BDO	RPUF-HDO	RPUF-DEG	RPUF-DPG
T_g_ (°C)	57.2	78.7	67.6	87.5

## Data Availability

Data are contained within the article.
